# Multiple Neuraminidase Containing Influenza Virus-like Particle Vaccines Protect Mice from Avian and Human Influenza Virus Infection

**DOI:** 10.3390/v14020429

**Published:** 2022-02-18

**Authors:** Hae-Ji Kang, Ki-Back Chu, Keon-Woong Yoon, Gi-Deok Eom, Jie Mao, Min-Ju Kim, Su-Hwa Lee, Eun-Kyung Moon, Fu-Shi Quan

**Affiliations:** 1 Department of Biomedical Science, Graduate School, Kyung Hee University, Seoul 02447, Korea; haedi1202@naver.com (H.-J.K.); kgang92@gmail.com (K.-W.Y.); ekd3910@naver.com (G.-D.E.); maojie@khu.ac.kr (J.M.); mj16441@naver.com (M.-J.K.); 2 Department of Medical Zoology, Kyung Hee University School of Medicine, Seoul 02447, Korea; ckb421@gmail.com (K.-B.C.); dltnghk228@nate.com (S.-H.L.); ekmoon@khu.ac.kr (E.-K.M.); 3 Medical Research Center for Bioreaction to Reactive Oxygen Species and Biomedical Science Institute, School of Medicine, Graduate School, Kyung Hee University, Seoul 02447, Korea

**Keywords:** avian influenza virus, virus-like particles, neuraminidase, heterosubtypic immunity

## Abstract

Avian influenza virus remains a threat for humans, and vaccines preventing both avian and human influenza virus infections are needed. Since virus-like particles (VLPs) expressing single neuraminidase (NA) subtype elicited limited heterosubtypic protection, VLPs expressing multiple NA subtypes would enhance the extent of heterosubtypic immunity. Here, we generated avian influenza VLP vaccines displaying H5 hemagglutinin (HA) antigen with or without avian NA subtypes (N1, N6, N8) in different combinations. BALB/c mice were intramuscularly immunized with the VLPs to evaluate the resulting homologous and heterosubtypic immunity upon challenge infections with the avian and human influenza viruses (A/H5N1, A/H3N2, A/H1N1). VLPs expressing H5 alone conferred homologous protection but not heterosubtypic protection, whereas VLPs co-expressing H5 and NA subtypes elicited both homologous and heterosubtypic protection against human influenza viruses in mice. We observed that VLP induced neuraminidase inhibitory activities (NAI), virus-neutralizing activity, and virus-specific antibody (IgG, IgA) responses were strongly correlated with the number of different NA subtype expressions on the VLPs. VLPs expressing all 3 NA subtypes resulted in the highest protection, indicated by the lowest lung titer, negligible body weight changes, and survival in immunized mice. These results suggest that expressing multiple neuraminidases in avian HA VLPs is a promising approach for developing a universal influenza A vaccine against avian and human influenza virus infections.

## 1. Introduction

Avian influenza outbreaks have caused vast economic losses throughout the world, with damage costs to the poultry industry being estimated to be in the billions [1]. However, the truly devastating aspect of avian influenza viruses requiring continuous monitoring is their potential for causing global pandemics via genetic reassortment and interspecies transmission. The H1N1 pandemic of 1918 was the first influenza outbreak of the 20th century and was responsible for the loss of over 50 million lives [2]. Sequence analysis results of the 1918 H1N1 influenza virus suggest that its nucleoprotein amino acid sequences were strikingly similar to those of avian influenza viruses currently found in wild birds [3,4]. Subsequent pandemics, which occurred in the years 1957 and 1968 by H2N2 and H3N2 influenza viruses, respectively, were reported to be of avian origin and underwent genetic reassortment with the circulating human H1N1 influenza virus [4]. To date, various strains of avian influenza viruses, including but not limited to H5N1 and H7N9, are reported to be circulating in many countries with occasional human infections [5]. The cumulative number of human clinical cases for highly pathogenic avian influenza H5N1 infections from the years 1997 to 2015 was reported to be around 900, with a fatality rate exceeding 50% [6]. Based on these historic findings, developing influenza virus vaccines that can protect against both avian and human influenza viruses is critically important.

Hemagglutinin (HA) is a key component of seasonal influenza vaccines and antibodies raised against this antigen can be protective [7,8]. Nevertheless, the selection pressure exerted by the host’s immune response paired with HA head domain plasticity forces the virus to undergo antigenic drift that renders the vaccines highly strain-specific [9]. Neuraminidase (NA) is another glycoprotein expressed by influenza viruses, which cleaves sialic acid groups on the host cell surface to enable viral particle release [10]. Several studies have demonstrated that while both HA and NA antigens undergo antigenic drift, this phenomenon occurs at a much slower rate for NA [11,12]. Despite this favorable aspect of NA antigens, which has a lower probability of escape mutations, NA antigens are often overlooked and underutilized in vaccines developed partly due to low antibody response as indicated by 18% mean seroconversion rate [10,13,14]. One possible solution is the use of virus-like particles (VLPs), a multivalent non-infectious particle completely devoid of replicative function in host organisms that can elicit high titers of long-lasting antibody responses [15]. Previous studies have shown that vaccinating animals with NA VLPs induced protection against homologous, heterologous, or heterosubtypic influenza viruses [7,16]. Although VLP vaccines expressing a single NA induced protection against homologous and heterologous influenza virus challenges, heterosubtypic immunity induction was limited and could not completely protect animals from challenge infections. Thus, constructing multivalent VLPs expressing different strains of NA antigens would be a potential strategy for inducing improved heterosubtypic cross-protection.

To date, studies reporting avian influenza vaccine-induced heterosubtypic protection against human influenza virus infections are extremely limited. Heterosubtypic protection against human influenza viruses was observed from peptide vaccines based on the M2 extracellular domain and also from inactivated virus vaccines [17,18]. On the contrary, immunization with the FDA-approved H5N1 (A/Indonesia/05/2005) influenza vaccine adjuvanted with AS03 failed to elicit neutralizing antibody response against H1N1 seasons influenza [19]. However, none of the aforementioned studies are based on NA antigens expressed on highly immunogenic vaccine platforms such as the VLPs, which have been documented to confer greater protection than whole inactivated influenza vaccines [18]. Here, we generated 8 different VLPs using the NA subtypes derived from avian influenza viruses. Heterosubtypic cross-protection against avian and human influenza viruses elicited by these VLPs expressing zero to three different NA subtypes was evaluated. We found that neuraminidase inhibition activity against the homologous virus (H5N1) and heterosubtypic virus (H3N2, H1N1) was positively correlated with the number of NA subtypes, with VLPs displaying all three NA subtypes conferring the strongest heterosubtypic protection against human influenza virus infections.

## 2. Materials and Methods

### 2.1. Mice, Cells, and Viruses

Female BALB/c mice were purchased from Nara Biotech (Seoul, Republic of Korea). All animal experimental procedures have been approved and conducted in accordance with the Kyung Hee University IACUC guidelines (permit ID: KHUASP(SE)-18-024). Spodoptera frugiperda 9 (Sf9) cells were used to generate recombinant baculoviruses (rBVs) and VLPs, while Madin-Darby canine kidney (MDCK) cells were used for microneutralization (MN) and influenza plaque assays as previously described [20,21]. Sf9 cells and MDCK cells were obtained from Dr. Richard W Compans at Emory University. Mouse adapted influenza viruses, A/chicken/Vietnam/G04/2004 (H5N1), A/Hong Kong/1/1968 (H3N2), or A/PR/8/34 (H1N1) were prepared as described [22,23].

### 2.2. Generation of Recombinant Baculovirus and Virus-Like Particles

Recombinant baculoviruses (rBVs) expressing the HA and NA proteins of avian influenza viruses were produced for VLP construction as previously described [24,25]. H5, N1, N6, and N8 genes were synthesized by GenScript (Piscataway, NJ, USA) and the gene sequences were derived from the following virus strains in respective order: A/chicken/Vietnam/G04/2004 (H5N1) for H5 and N1, A/environment/Korea/W544/2016 (H5N6) for N6, and A/Baikal teal/Korea/H41/2014 (H5N8) for N8. Sf9 cells were co-infected with rBVs expressing M1, H5, N1, N6, or N8 at 0.2 MOI. A total of 8 different VLPs expressing either HA alone or both HA and NA surface antigens on top of the matrix 1 (M1) core protein were generated and their compositions are as follows: HA VLPs, H5N1 VLPs, H5N6 VLPs, H5N8 VLPs, H5N1N6 VLPs, H5N1N8 VLPs, H5N6N8 VLPs, and H5N1N6N8 VLPs. Sf9 cell culture supernatants were harvested 3 days post-infection (dpi) and centrifuged at 6000 rpm, 30 min, 4 °C to remove cellular debris. After centrifuging the supernatants at 30,000 rpm, 20 min, 4 °C, sedimented VLPs were purified through a sucrose gradient and stored at −80 °C until use.

### 2.3. Characterization of VLPs

VLPs were characterized using western blot and either HA or NA activity assays as previously described [7]. For western blots, HA antigen expressions were detected using influenza H5N1-infected mice sera (1:1000 dilution), while rabbit anti-NA monoclonal antibody (HCA-2, 1:10,000 dilution) was used to confirm the expression of NA antigens [26]. Hemagglutination assay was performed to assess HA activity, and neuraminidase activities of VLPs were measured using the Amplex Red neuraminidase assay kit (Invitrogen, Carlsbad, CA, USA). Transmission electron microscopy (TEM) (JEOL 2100, JEOL USA, Inc.; Peabody, MA, USA) was used to observe the morphology of the VLPs as described previously [27]. Stained grids were observed under TEM and images confirming successful construction of VLPs were captured.

### 2.4. Immunization and Challenge Infection

Seven-week-old female BALB/c mice (*n* = 6 per group) were used for immunization and challenge infection studies. Mice were intramuscularly immunized twice with 10 μg of each VLPs in 100 uL of PBS at a 4-week interval. Blood samples were collected from each mouse 4 weeks after prime and boost immunizations. Four weeks after the second immunization, isoflurane-anesthetized mice were intranasally challenged infected with lethal doses (5LD50) of H5N1, H3N2, or H1N1 influenza viruses in 50 uL of PBS, and sera were collected at 4 dpi. Naïve mice and unimmunized infection control (Naïve+cha) were used as control groups. All mice were observed daily to monitor bodyweight changes and mortality. In accordance with the Institutional Animal Care and Use Committee (IACUC) guideline, 25% body weight loss was determined to be the humane intervention point, and mice reaching this endpoint were humanely euthanized.

### 2.5. Antibody Response, Virus Titer, and Cytokine Response

Enzyme-linked immunosorbent assay (ELISA) was performed to determine influenza virus-specific antibody responses as described in [24]. Inactivated H5N1, H3N2, and H1N1 influenza viruses (4 μg/mL) were used as coating antigens and antigen-specific IgG, IgG1, IgG2a, and IgA antibodies from sera and the homogenized lung extracts were assessed [26]. Inflammatory cytokines interferon-gamma (IFN-γ) and interleukin-6 (IL-6) from the lung extracts were measured using ELISA kits as described previously [24,28]. Lung virus titers were determined by plaque assay using MDCK cells as described in [23].

### 2.6. Microneutralization Activity

For microneutralization activity assessment, 100 TCID¬50 per well of H5N1, H3N2, and H1N1 influenza viruses were determined and prepared as previously described [29,30]. Briefly, immune sera collected after boost immunization were incubated with receptor destroying enzyme (RDE) at 1:3 ratio for 18 h, 37 °C, and subsequently heat-inactivated for 30 min at 56 °C. Inactivated sera were reacted with 100 TCID50 influenza viruses for 1 h at 37 °C, 5% CO_2_, and the samples were incubated with MDCK cells for 18 h at 37 °C, 5% CO_2_. After fixing the cells with cold acetone fixative, cells were sequentially incubated with anti-influenza A NP monoclonal antibody and horseradish peroxidase-conjugated IgG. O-phenylenediamine dihydrochloride (OPD) substrate was used and OD490 nm values were measured to determine the microneutralization titers. Virus neutralizing anti-body 50% titers were calculated using the following equation: (average OD of virus control wells—average OD of cell control)/2.

### 2.7. Neuraminidase Inhibition (NAI) Analysis

Enzyme-linked lectin assay (ELLA) was performed using the sera of boost-immunized mice to measure neuraminidase inhibition as previously described [7]. Briefly, 96 well plates were coated with 100 uL of fetuin (25 ug/mL) in carbonate coating buffer and incubated overnight at 4 °C. After blocking, wells were incubated with serially diluted immune sera and highly active influenza virus mixture at 37 °C for 2 h. Upon incubation with the peroxidase-conjugated peanut agglutinin (1 ug/mL), tetramethylbenzidine substrate was added and OD 490 nm was measured to determine NAI. The 50% inhibition percentage was defined as the serum concentration resulting in at least 50% inhibition compared to the virus-only control.

### 2.8. Flow Cytometry

Lung tissues of challenge-infected mice were collected at 4 dpi, and single-cell suspensions were used to analyze CD4+ T cell, CD8+ T cell, germinal center-like (GC) cell, and B cell populations [7,24]. Harvested cells were stimulated with H5N1 and H3N2 inactivated virus antigens (0.5 ug/mL) for 5 h at 37 °C before staining with anti-CD3 (PeCy 5), CD4 (FITC), CD8 (PE), B220 (FITC), GL7(PE), CD19 (PeCy5), and IgD (PE) fluorophore-conjugated antibodies (BD Biosciences, San Diego, CA, USA) as described previously [31]. Cell acquisition and analyses were performed using the Accuri C6 flow cytometer and the C6 analysis software, respectively (BD Biosciences, San Diego, CA, USA).

### 2.9. Statistical Analysis

Statistical significances were determined by one-way analysis of variance (ANOVA) with Tukey’s multiple-comparison tests using the Prism 5 software (GraphPad Software, Inc., San Diego, CA, USA). Direct comparison between groups were indicated using lines and statistical significance was denoted by asterisks, where * *p* < 0.05, ** *p* < 0.01, and *** *p* < 0.001.

## 3. Results

### 3.1. Production of Influenza VLPs Containing Influenza HA, NA, and M1

**Figure 1:**
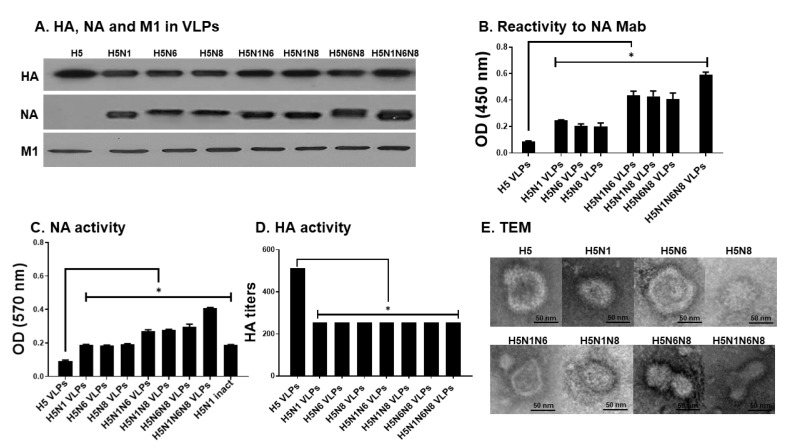
Characterization of influenza VLP vaccines. (**A**) The expression of NA, HA, and M1 proteins on VLPs was determined by western blot probed with rabbit HCA-2 monoclonal antibody, anti-H5N1 polyclonal antibody, and M1 monoclonal antibody. A total of 5 μg of each influenza H5, H5N1, H5N6, H5N8, H5N1N6, H5N1N8, H5N6N8, and H5N1N6N8 VLPs were loaded in each lane. (**B**) The reactivity of VLPs to NA mAb (HCA-2) was measured by ELISA. (**C**) The activity of NA proteins in VLPs was determined by the Amplex Red neuraminidase assay kit. (**D**) The activity of HA protein in VLPs was determined by hemagglutinin activity assay using cRBC. (**E**) Influenza H5, H5N1, H5N6, H5N8, H5N1N6, H5N1N8, H5N6N8, and H5N1N6N8 VLPs were observed under a transmission electron microscope. All data are expressed as mean ± SD (* *p* < 0.05).

Insect cell-derived VLPs were purified and characterized via western blot and ELISA. Successful expression of HA, NA, and M1 proteins in the VLPs was confirmed by western blot (Figure 1A). To confirm whether multiple NA expressions on the VLPs were proportional to increased reactivity to NA monoclonal antibody, ELISA was performed. Expectantly, the optical density readings were correlated with the number of NA subtype expressions on the VLPs (Figure 1B). Similar to this finding, additional NA expressions were associated with increased functional NA activity (Figure 1C). Hemagglutination activity of the H5-expressing VLPs was determined by reacting 5 μg of VLPs with the chicken red blood cells (cRBCs). Hemagglutination (HA) activity normalized to per μg of VLPs was 100 for H5 VLPs, which was 2 fold greater than those of H5N1 VLPs, H5N6 VLPs, H5N8 VLPs, H5N1N6 VLPs, H5N1N8 VLPs, H5N6N8 VLPs, and H5N1N6N8 VLPs (Figure 1D). TEM images confirmed the proper formation of VLPs resembling the morphology of influenza viruses, with extensive antigen spikes decorating the surface of the M1 protein (Figure 1E).

### 3.2. VLPs Vaccination Induced H5N1 or H3N2 Virus-Specific Antibody Responses in Sera and Lungs

**Figure 2:**
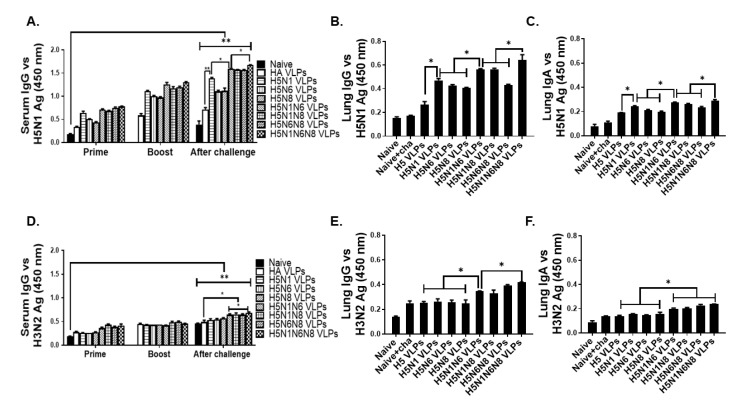
H5N1 and H3N2 virus-specific antibody responses in serum and lung. Mice (*n* = 6) were intramuscularly immunized twice at 4-week intervals with the 8 following VLPs: HA VLPs, H5N1 VLPs, H5N6 VLPs, H5N8 VLPs, H5N1N6 VLPs, H5N1N8 VLPs, H5N6N8 VLPs, and H5N1N6N8 VLPs. Blood samples were collected on week 4 after prime and boost, and 4 days after challenge infections with a lethal dose of H5N1 or H3N2. Lungs were collected and homogenized 4 days post-challenge infections. H5N1 influenza virus-specific IgG (**A**,**B**) and IgA (**C**) antibody responses in the lungs and sera, and H3N2 influenza virus-specific IgG (**D**,**E**) and IgA (**F**) antibody responses in the lungs and sera were determined by ELISA. All data are expressed as mean ± SD (* *p* < 0.05, ** *p* < 0.01).

Groups of mice were intramuscularly immunized with VLPs by a prime-boost regimen, and H5N1 and H3N2-specific antibody responses were evaluated from sera and lungs. To confirm successful vaccine-induced immunity inductions, H5N1 virus-specific antibody responses were assessed after prime, boost, and post-challenge infections. Increasing the number of VLP immunizations was positively correlated with enhanced H5N1-specific IgG response, irrespective of the VLP antigen formulations (Figure 2A). At 4 dpi, heightened pulmonary IgG and IgA responses were observed (Figure 2B,C). Compared to VLPs expressing HA alone, VLPs co-expressing NA antigens elicited higher levels of virus-specific antibody responses in sera and lungs of mice post-challenge. Incorporating multiple NA antigens into VLPs was associated with marginally increased virus-specific IgG responses. To demonstrate that the VLPs were cross-protective, heterosubtypic immunity against the H3N2 virus was evaluated by assessing the H3N2-specific IgG and IgA responses. While VLPs failed to elicit significant virus-specific IgG responses in sera even after two immunizations, significant differences were noticeable after challenge infection especially between HA VLPs and VLPs expressing multiple NA subtypes (Figure 2D). Antibody induction by VLPs co-expressing a single NA subtype in addition to the HA antigen was negligible in comparison to the naïve and HA VLP controls. Such findings were also observed from lung IgG and IgA responses after challenge infection. Though the differences were non-existent between the controls and single NA VLPs, VLPs displaying multiple NA subtypes significantly enhanced the H3N2-specific IgG and IgA responses (Figure 2E,F). Expectedly, the most potent pulmonary antibody induction was observed from the lungs of mice immunized with the H5N1N6N8 VLPs. These results indicated that the levels of IgG antibodies in sera and IgG and IgA antibodies in lungs were increased following NA incorporation into HA VLPs.

### 3.3. VLPs Vaccination Induced H5N1 or H3N2 Virus-Specific Antibody Responses in Sera and Lungs

**Figure 3:**
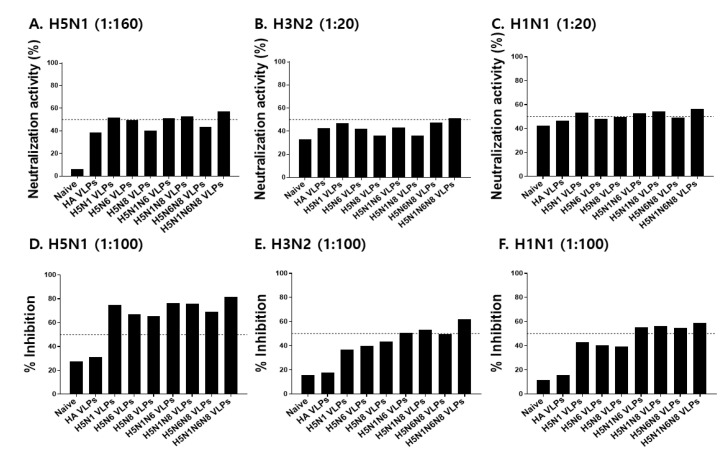
Viral neutralizing activity and neuraminidase inhibition activity against H5N1, H3N2, and H1N1 influenza viruses. Mouse sera at 4 weeks after boost immunization were used at various serum dilutions to determine viral neutralizing activity and neuraminidase inhibition activity. Microneutralization activities against H5N1 (**A**), H3N2 (**B**), or H1N1 (**C**) influenza viruses were determined in MDCK cells seeded in 96 well plates. NA inhibition activity was determined by an enzyme-linked lectin assay against H5N1 (**D**), H3N2 (**E**), or H1N1 (**F**) influenza viruses. The dotted line indicates 50% of virus-neutralizing activity and 50% of neuraminidase inhibition activity.

To confirm the functionality of the induced antibodies, microneutralizing activity, and neuraminidase inhibition activity against H5N1, H3N2, and H1N1 were assessed. Our results demonstrated that neutralizing titer against H5N1 was positively correlated with the number of NA antigens expressed on the VLP surface. VLPs expressing all three N1, N6, and N8 subtypes exhibited a neutralizing titer of 320, while those expressing either two or one NA subtypes exhibited a titer of 160 and between 80 and 160, respectively. The neutralizing titer of HA VLPs, which lack NA antigen expression, was determined to be 80 (Figure 3A). To validate the heterosubtypic protection induced by the antibodies, neutralizing activity against H3N2 and H1N1 was evaluated. Nevertheless, VLP immunization-induced antibodies demonstrated weak neutralizing activities against the two virus subtypes (Figure 3B,C). To further discern the relationship between multiple NA subtype expressions on the VLPs and virus inhibition, NA inhibition activities were assessed. The Highest NA inhibition against H5N1 was observed from VLPs expressing 3 different NA subtypes, with potent inhibition occurring at 1:100 serum dilution. Inhibitory activities of HA VLPs were comparable to those of naïve control (Figure 3D). A similar trend was observed against heterosubtypic challenge infection. VLPs coexpressing all three N1, N6, and N8 subtypes demonstrating potent inhibition rates against H3N2 and H1N1 at 1:100 serum dilutions (Figure 3E,F). These results indicate that while increasing NA antigen contents in the VLPs play a critical role in NA inhibition against both avian and human influenza viruses, their contribution to virus neutralization is negligible.

### 3.4. VLP Vaccines Elicited H5N1 or H3N2 Virus-Specific CD4+ and CD8+ T Cell Immune Responses in the Lung

**Figure 4:**
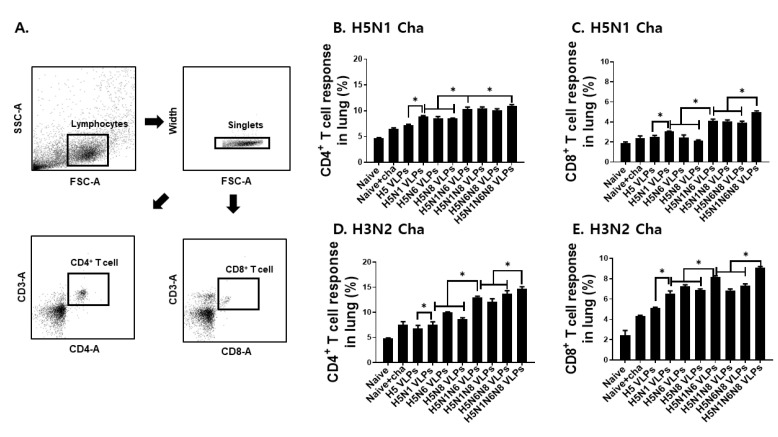
CD4+ T cell and CD8+ T cell immune responses in the lungs upon challenge infection with H5N1 or H3N2 influenza viruses. BALB/c mice were intramuscularly immunized twice with the VLPs. Lung samples were collected at 4 days post-challenge infections with H5N1 or H3N2 influenza viruses. Lung cells were gated using the strategy depicted above (**A**) to determine CD4+ T cell (**B**,**D**) and CD8+ T cell (**C**,**E**) responses. Immune cells collected from the lungs were stimulated with inactivated viruses (0.5 μg /mL) from H5N1 or H3N2. The lung cells were stained with anti-CD3, CD4, CD8 and the populations were determined by flow cytometry analysis. All data are expressed as mean ± SD (* *p* < 0.05).

The lung is an important organ for evaluating T cell response upon influenza virus infection. Four days after challenge infection with H5N1 or H3N2, murine lungs were isolated and the immune responses of CD4+ and CD8+ T cells were confirmed from total cells. Lung cells were gated appropriately to determine CD4+ and CD8+ T cell populations (Figure 4A). Compared to HA VLPs or VLPs with a single NA subtype, immunization with the VLPs displaying multiple NA subtypes was associated with enhanced CD4+ (Figure 4B,D) and CD8+ (Figure 4C,E) T cell responses in the lungs of mice. CD4+ and CD8+ T cell responses for VLPs expressing a single NA subtype were 8.6% and 6.9%, whereas the T cell responses induced upon immunization with the VLPs expressing 2 NA subtypes were 12.9% and 7.4%, respectively. The strongest T cell response induction was observed from mice immunized with the VLPs expressing all three N1, N6, and N8 subtypes, which resulted in 14.7% and 9.1% CD4+ and CD8+ T cell responses, respectively. Interestingly, mice immunized with the VLPs expressing at least 2 different NA subtypes elicited 1.3 fold higher CD4+ and CD8+ T cell inductions when challenge infected with H3N2 than the H5N1 influenza virus.

### 3.5. VLPs Vaccine Elicited H5N1 or H3N2 Viruses-Specific Germinal Center-Like Cell and B Cells Immune Responses in the Lung

**Figure 5:**
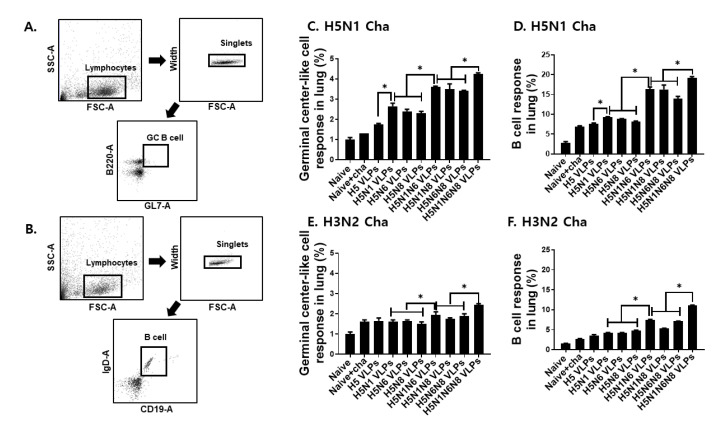
Mice were immunized twice with VLPs and challenge infected with a lethal dose of H5N1 or H3N2 influenza viruses. Lungs were isolated from mice 4 days post-challenge infection and lung cells were stained with B220, GL7, CD19, and IgD. Single cell populations of lung cells were gated to assess proliferation of GC-like B cells and B cells (**A**,**B**). Germinal center-like cell (**C**,**E**) and B cell (**D**,**F**) populations were determined by flow cytometry. All data are expressed as mean ± SD (* *p* < 0.05).

Mice immunized with the VLPs were challenge infected with either H5N1 or H3N2 influenza viruses, and the germinal center-like cell and B cell immune responses in the lungs were determined. Lung cells were gated to assess GC-like B cell and B cell populations from all groups (Figure 5A,B). As seen in Figure 5, GC B cell (Figure 5C,E) and B cell (Figure 5D,F) inductions in the lungs were largely influenced by NA expressions. Differences between unimmunized, HA VLP, and single NA containing HA VLPs were negligible. However, incorporating two or more NA subtypes resulted in greater GC B and B cell inductions against both H5N1 and H3N2 challenge infections.

### 3.6. VLPs Vaccination Significantly Reduced Inflammatory Cytokine Response and Lung Virus Titers

**Figure 6:**
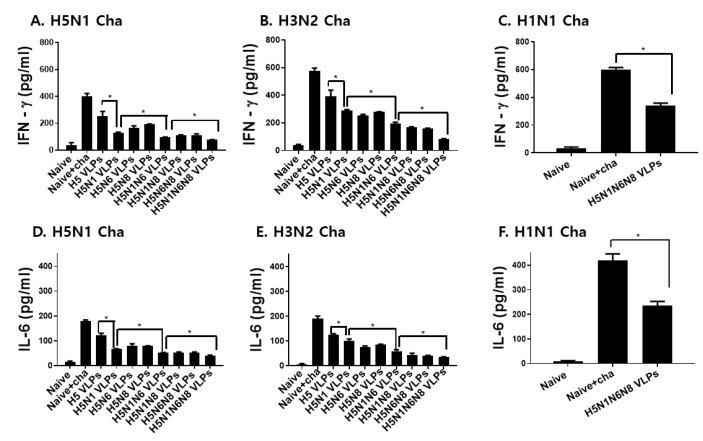
Lung inflammatory cytokine responses upon influenza viruses challenge infection. Mice were immunized twice with the VLPs and were challenged with H5N1, H3N2, or H1N1 influenza viruses. On day 4 post-challenge infections, lungs were harvested and the lung inflammatory cytokines IFN-γ (**A**–**C**) and IL-6 (**D**–**F**) were determined by cytokine ELISA kits. All data are expressed as mean ± SD (* *p* < 0.05).

**Figure 7:**
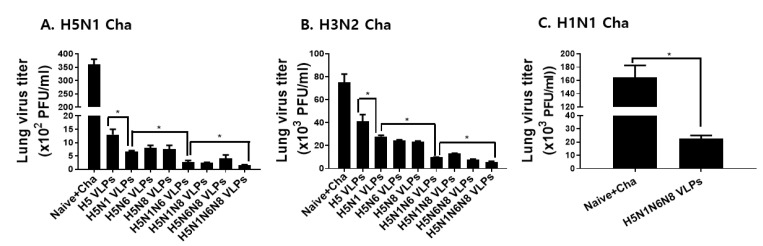
Lung virus titers upon influenza viruses challenge infection. VLP immunized mice were challenge infected with H5N1 (**A**), H3N2 (**B**), or H1N1 (**C**) influenza viruses. On day 4, lung samples were obtained and the lung virus titers were determined using MDCK cells. All data are expressed as mean ± SD (* *p* < 0.05).

Inflammatory cytokine response and lung virus loads upon challenge infections are important parameters in the vaccine efficacy evaluation. As shown in Figure 6 and Figure 7, inflammatory cytokine production and lung virus titers were inversely proportional to the amount of NA antigens displayed on the VLP surface. Coexpressing H5 and a single NA subtype substantially diminished the IFN-γ production in the lungs of mice, which were further reduced with additional NA expressions against both avian and human influenza viruses (Figure 6A–C). A similar trend was observed for IL-6, whose production was negatively correlated with the number of NA antigens (Figure 6D–F). To affirm that the reduced inflammatory response was the result of lessened lung viral loads, virus titers in the lungs of mice were determined. Consistent with the inflammatory cytokine results, lung virus titers following challenge infections with H5N1, H3N2, and H1N1 were inversely proportional to the number of NA antigens displayed on the VLPs (Figure 7A–C). These results indicated that NA is critical for reducing lung inflammatory cytokines and lung viral loads.

### 3.7. VLPs Vaccine Efficacy against H5N1, H3N2, and H1N1 Virus Challenge Infections

**Figure 8:**
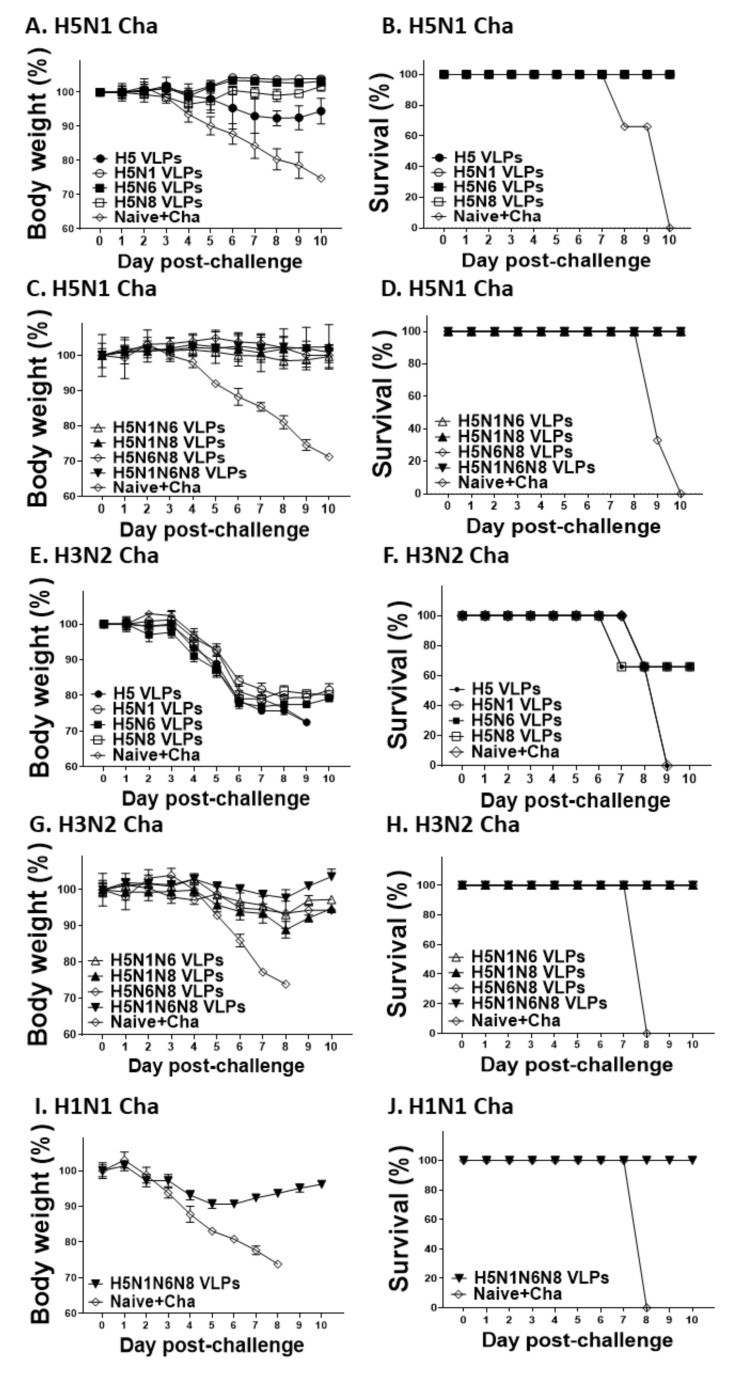
Protective efficacy upon influenza viruses challenges infection. VLP immunized mice were challenge infected with lethal doses of H5N1, H3N2, or H1N1 influenza viruses 4 weeks after boost immunization. Mice were monitored daily to record body weight changes after H5N1 (**A**,**C**), H3N2 (**E**,**G**), and H1N1 (**I**) infections, and their survival rates post-infection with H5N1 (**B**,**D**), H3N2 (**F**,**H**) and H1N1 influenza virus (**J**).

Mice immunized with VLPs were challenge infected with H5N1, H3N2, or H1N1 influenza viruses. Challenge infection with the H5N1 virus incurred negligible bodyweight loss in VLP-immunized mice, with HA VLP being the sole exception as substantial body weight loss was observed (Figure 8A,B). Upon H3N2 virus challenge infection, all of the mice immunized with the VLPs expressing either HA alone or HA with a single NA subtype underwent drastic body weight loss (Figure 8C). Co-expression of a single NA subtype was sufficient to confer partial protection against H3N2, as indicated by 66% survival compared to 0% survival observed from HA VLP-immunized mice (Figure 8D). Additional NA expression in the VLPs was associated with lessened bodyweight loss and enhanced survival. Bodyweight changes in mice immunized with the VLPs expressing two NA subtypes were less than 10% and all of the immunized mice survived. For three NA-expressing VLPs, bodyweight reductions following H3N2 challenge infection were not detected and 100% survival was observed (Figure 8E,F). To further confirm that avian influenza VLP vaccines can confer protection against other prominent human influenza viruses, mice immunized with the three NA-expressing VLPs were challenged with the H1N1 influenza virus. Our results demonstrated that avian influenza VLPs displaying 3 different NA subtypes also protected mice against H1N1. The body weight changes incurred post-challenge with H1N1 was less than 10% with a 100% survival rate, indicating that multiple NA expressions were critical for protection against heterosubtypic virus infections (Figure 8G,H).

## 4. Discussion

Influenza virus antigens are constantly evolving, with frequent antigenic drift contributing to annual outbreaks and occasional pandemics. Given these present circumstances, universal influenza vaccine development is of utmost importance to prevent a pandemic should it occur in the near future. The first clinical case study reporting human infection with the avian influenza virus H5N1 was in Hong Kong 1997 [19]. Since then, novel avian influenza virus infections have surged in humans, including H5N6, H6N1, H7N9, H10N8 with some of them being fatal [32,33,34]. Ideally, the universal influenza vaccine should provide complete protection irrespective of subtype or changes to antigens introduced through mutations. Here, we demonstrated that avian influenza VLP vaccines confer homologous and heterosubtypic protection against both avian and human influenza viruses, with their efficacies being dependent on the number of neuraminidase subtypes. Influenza virus NA-based immunity is well known for contributing to broad protection [10,16]. A wide array of influenza VLP vaccine studies reported the presence of heterosubtypic immunity induced by NA antigens derived from multiple human influenza viruses, although the heterosubtypic protection conferred was limited [7,16,20]. In line with this notion, mice immunized with the VLPs expressing the HA and NA of 1918 pandemic influenza underwent dramatic bodyweight loss and eventually perished following H5N1 challenge infection, which was identical to the fate of negative control mice immunized with the HIV VLPs [20]. Immunizing mice with VLPs displaying the NA subtypes derived from the 2009 pandemic H1N1 strain did not result in their death nor drastic bodyweight loss upon challenge infection with A/H3N2 and A/H5N1, which can be attributed to the low challenge infection dosage [7]. Another N1 VLPs derived from 2009 pandemic H1N1 reported that VLPs vaccination in mice exhibited heterosubtypic protection against A/Philippines/82 (H3N2) influenza virus, although substantial body weight loss was observed (23.5%) [16]. While none of these studies were based on immunization with NA VLPs based on avian influenza strains, the aforementioned works indicated that the immunogenicity of NA VLPs needs to be improved to induce heterosubtypic protection.

Consistent with these previous findings, heterosubtypic immunity from NA VLPs were observed in our study. Intriguingly, our findings indicated that the degree of heterosubtypic protection appeared to be dependent on the number of different NA subtypes being displayed. VLPs expressing all three N1, N6, and N8 subtypes elicited the highest NAI, thus indicating that three avian NA incorporated HA VLPs can protect mice from human influenza virus infections. In our study, the highest level of heterosubtypic protection, evaluated by measuring human influenza virus-specific antibody responses in sera and lungs, germinal center-like cell responses, T cell responses, and inflammatory cytokine production in the lungs, was demonstrated by HA VLPs displaying all 3 NA subtypes.

Formulating vaccines with NA could be the key to the successful development of universal influenza vaccines. Currently, influenza vaccines are predominantly based on HA which specifically induces antibodies against the HA antigen, and as such, optimizing future vaccines by incorporating NA should be considered. When considered at molar levels, NA antigens are thought to be the most immunogenic of all the influenza proteins despite being less abundant than HA proteins [35]. Recently, Chen et al. [30] demonstrated that NA-reactive B cell induction through natural influenza virus infection elicited antibodies that confer broad protection against both human and avian influenza strains, as well as the oseltamivir-resistant influenza variants. Consistent with this finding, VLPs expressing NA and M2e elicited cross-reactive CD8+ T cells and induced heterologous protection, whereas VLPs expressing HA antigen only conferred homologous protection [36]. Our findings also support this notion as increasing the expression of different NA subtypes promoted CD8+ T cell responses in the lungs of mice. Also, immunization with influenza VLPs induces antigen-specific B cells, which play a dominant role in CD4+ T cell proliferation and expression of associated cytokines [37]. These results were also observed from our study, as indicated by the increases in lung B cell and CD4+ T cell populations.

In summary, we generated multiple avian influenza NA-expressing VLPs which conferred homologous and heterosubtypic protection. In particular, mice immunized with the VLPs expressing 3 NA subtypes induced the highest level of NAI and immune responses in the lungs upon challenge infection with the human influenza viruses. Our data revealed that incorporating multiple NA subtypes into vaccine platforms contributes to mounting robust immune response that broadly protects against both homologous and heterosubtypic virus challenge infections, providing new insight for developing effective universal influenza VLPs vaccines. This approach could be crucial to preventing an influenza outbreak in both birds and humans, irrespective of the virus origin and its subtype. Overall, the vaccine design strategy presented herein has enormous potential, and further assessment in model organisms that better reflect humans should be conducted.

## Data Availability

Data supporting the findings of this study are contained within the article.
